# Empirical software metrics for benchmarking of verification tools

**DOI:** 10.1007/s10703-016-0264-5

**Published:** 2017-01-10

**Authors:** Yulia Demyanova, Thomas Pani, Helmut Veith, Florian Zuleger

**Affiliations:** TU Wien, Karlsplatz 13 1040 Vienna, Austria

**Keywords:** Software verification, Software metrics, Machine learning, Algorithm portfolio

## Abstract

We study empirical metrics for software source code, which can predict the performance of verification tools on specific types of software. Our metrics comprise variable usage patterns, loop patterns, as well as indicators of control-flow complexity and are extracted by simple data-flow analyses. We demonstrate that our metrics are powerful enough to devise a machine-learning based portfolio solver for software verification. We show that this portfolio solver would be the (hypothetical) overall winner of the international competition on software verification (SV-COMP) in three consecutive years (2014–2016). This gives strong empirical evidence for the predictive power of our metrics and demonstrates the viability of portfolio solvers for software verification. Moreover, we demonstrate the flexibility of our algorithm for portfolio construction in novel settings: originally conceived for SV-COMP’14, the construction works just as well for SV-COMP’15 (considerably more verification tasks) and for SV-COMP’16 (considerably more candidate verification tools).

## Introduction

The success and gradual improvement of software verification tools in the last two decades is a multidisciplinary effort—modern software verifiers combine methods from a variety of overlapping fields of research including model checking, static analysis, shape analysis, SAT solving, SMT solving, abstract interpretation, termination analysis, pointer analysis etc.

The mentioned techniques all have their individual strengths, and a modern software verification tool needs to pick and choose how to combine them into a strong, stable and versatile tool. The trade-offs are based on both technical and pragmatic aspects: many tools are either optimized for specific application areas (e.g. device drivers), or towards the in-depth development of a technique for a restricted program model (e.g. termination for integer programs). Recent projects like CPA [6] and FrankenBit [22] have explicitly chosen an eclectic approach which enables them to combine different methods more easily.

There is growing awareness in the research community that the benchmarks in most research papers are only useful as proofs of concept for the individual contribution, but make comparison with other tools difficult: benchmarks are often manually selected, handcrafted, or chosen a posteriori to support a certain technical insight. Oftentimes, neither the tools nor the benchmarks are available to other researchers. The annual *international competition on software verification* (SV-COMP, since 2012) [3–5, 12–14] is the most ambitious attempt to remedy this situation. Now based on more than 6600 C source files, SV-COMP has a diverse and comprehensive collection of benchmarks available, and is a natural starting point for a more systematic study of tool performance.

In this paper, we demonstrate that the competition results can be explained by intuitive metrics on the source code. In fact, the metrics are strong enough to enable us to construct a portfolio solver which would (hypothetically) win SV-COMP 2014 [12], 2015 [13], and 2016 [14]. Here, a portfolio solver is a software verification tool which uses heuristic preprocessing to select one of the existing tools [21, 26, 34].

**Table 1:** Sources of complexity for 4 tools participating in SV-COMP’15, marked with + / – / n/a when supported/not supported/no information is available

Source of complexity	CBMC	Predator	CPAchecker	SMACK	Corresp. feature
Unbounded loops	–	n/a	n/a	–	$\mathcal {L}^{\mathrm{SB}}, \mathcal {L}^{\mathrm{ST}}, \mathcal {L}^{\mathrm{simple}}, \mathcal {L}^{\mathrm{hard}}$
Pointers	$+$	$+$	$+$	$+$	PTR
Arrays	$+$	–	n/a	$+$	ARRAY_INDEX
Dynamic data structures	n/a	$+$	n/a	$+$	PTR_STRUCT_REC
Non-static pointer offsets	–	$+$	n/a	n/a	OFFSET
Non-static size of heap-allocated memory	$+$	$+$	n/a	n/a	ALLOC_SIZE
Pointers to functions	$+$	n/a	n/a	n/a	$m_\mathrm{fpcalls}, m_\mathrm{fpargs}$
Bit operations	$+$	–	$+$	–	BITVECTOR
Integer variables	$+$	$+$	$+$	$+$	SCALAR_INT
Recursion	–	–	–	$+$	$m_\mathrm{reccalls}$
Multi-threading	$+$	–	–	–	THREAD_DESCR
External functions	$+$	–	n/a	n/a	INPUT
Structure fields	$+$	$+$	n/a	$+$	STRUCT_FIELD
Big CFG ($\ge$100 KLOC)	$+$	n/a	n/a	$+$	$m_\mathrm{cfgblocks}, m_\mathrm{maxindeg}$

Extracted from the competition report [2] and tool papers [10, 19]

Of course it is pointless to let a portfolio solver compete in the regular competition (except, maybe in a separate future track), but for anybody who just wants to verify software, it provides useful insights. As an approach to software verification, portfolio solving brings interesting advantages:

1. A portfolio solver *optimally uses available resources*.
  While in theory one may run all available tools in parallel, in practice the cost of setup and computational power makes this approach infeasible. A portfolio predicts the *n* tools it deems best-suited for the task at hand, allowing better resource allocation.
2. It can *avoid incorrect results of partially unsound tools*.
  Practically every existing software verification tool is partially incomplete or unsound. A portfolio can recognize cases in which a tool is prone to give an incorrect answer, and suggest another tool instead.
3. Portfolio solving allows us to *select between multiple versions of the same tool*.
  A portfolio is not only useful in deciding between multiple independent tools, but also between the same tool with different runtime parameters (e.g. command-line arguments).
4. The portfolio solver *gives insight into the state-of-the-art* in software verification.
  As argued in [43], the state-of-the-art can be set by an automatically constructed portfolio of available solvers, rather than the *single best solver* (e.g. a competition winner). This accounts for the fact that different techniques have individual strengths and are often complementary.

To choose the software metrics describing our benchmarks, we consider the zoo of techniques discussed above along with their target domains, our intuition as programmers, as well as the tool developer reports in their competition contributions. Table 1 exemplarily summarizes these reports for tools CBMC, Predator, CPAchecker and SMACK: the first column gives obstacles the tools’ authors identified, the following columns show whether the feature is supported by respective tool, and the last column references the corresponding metrics, which we introduce in Sect. 2. The obtained metrics are naturally understood in three dimensions that we motivate informally first:

1. *Program variables* Does the program deal with machine or unbounded integers? Are the ints used as indices, bit-masks or in arithmetic? Dynamic data structures? Arrays? Interval analysis or predicate abstraction?
2. *Program loops* Reducible loops or goto programs? FOR-loops or ranking functions? Widening, loop acceleration, termination analysis, or loop unrolling?
3. *Control flow* Recursion? Function pointers? Multithreading? Simulink-style code or complex branching?

Our hypothesis is that precise metrics along these dimensions allow us to predict tool performance. The challenge lies in identifying metrics which are predictive enough to understand the relationship between tools and benchmarks, but also simple enough to be used in a preprocessing and classification step. Sections 2.1, 2.2 and 2.3 describe metrics which correspond to the three dimensions sketched above, and are based on simple data-flow analyses.

Our algorithm for building the portfolio is based on machine learning using *support vector machines* (SVMs) [8, 16] over these metrics. Section 3 explains our approach for constructing the portfolio.

Finally, we discuss our experiments in Sect. 4. In addition to previous results on SV-COMP’14 and ’15 in [17], we apply our portfolio construction to new data from SV-COMP’16, which has recently become available. As before, our portfolio is the hypothetical winner. As the underlying machine learning problem is becoming harder from year to year (considerably more verification tasks and candidate tools), this showcases the overall flexibility of our approach. We highlight the major differences between the three SV-COMP editions ’14–’16 in Sect. 4.1.

**Fig. 1:**
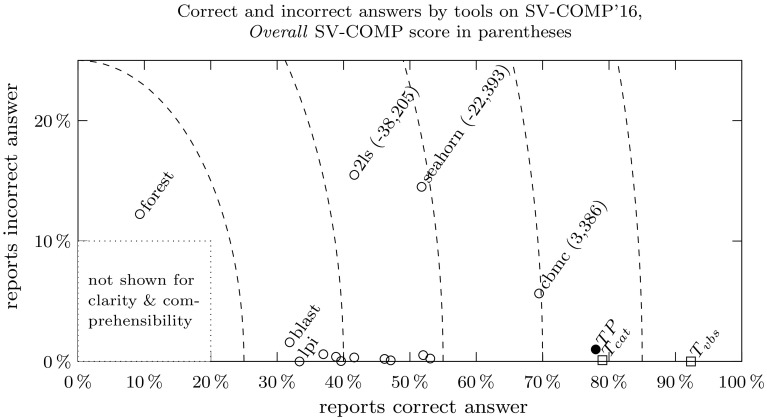
Decisiveness-reliability plot for SV-COMP’16. The *horizontal axis* gives the percentage of correct answers *c*, the *vertical axis* the number of incorrect answers *i*. *Dashed lines* connect points of equal decisiveness $c+i$. The *Overall* SV-COMP score is given (if available) in parentheses

Figure 1 depicts our results on SV-COMP’16: Our tool $\mathcal {TP}$ (identified by $\bullet$ $\textit{TP}$) is the overall winner and outperforms all other tools (identified by a $\circ$). Section 4 contains a detailed discussion of our experiments.

While portfolio solvers are important, we also think that the software metrics we define in this work are interesting in their own right. Our results show that categories in SV-COMP have characteristic metrics. Thus, the metrics can be used to (1) characterize benchmarks not publicly available, (2) understand large benchmarks without manual inspection, (3) understand presence of language constructs in benchmarks.

Summarizing, in this paper we make the following contributions:

- We define software metrics along the three dimensions – program variables, program loops and control flow – in order to capture the difficulty of program analysis tasks (Sect. 2).
- We develop a machine-learning based portfolio solver for software verification that learns the best-performing tool from a training set (Sect. 3).
- We experimentally demonstrate the predictive power of our software metrics in conjunction with our portfolio solver on the software verification competitions SV-COMP’14, ’15, and ’16 (Sect. 4).

This paper is an extended version of our previous work [17], which additionally covers:

- We apply the portfolio construction from [17] to SV-COMP’16 and report on the results. In particular, our portfolio is again winning the *Overall* category (Sect. 4).
- We include detailed results tables for our experiments on SV-COMP’14–’16. (Sect. 4).
- We extend our experiments on memory usage and runtime as a tie breaker in our tool selection algorithm (Sect. 3.3).
- We extend the description of loop patterns, which have only been motivated in the conference article (Sect. 2.2).
- We improve the explanation of support vector machines for non-linearly separable data, motivating their use in our portfolio construction (Sect. 3.1).

## Source code metrics for software verification

We introduce program features along the three dimensions—*program variables*, *program loops* and *control flow*—and describe how to derive corresponding metrics. Subsequent sections demonstrate their predictive power: In Sect. 3 we describe a portfolio solver for software verification based on our metrics. In Sect. 4 we experimentally demonstrate the portfolio’s success, thus attesting the descriptive and predictive power of our metrics and the portfolio.

### Variable role based metrics

The first set of features we consider are *patterns of variable usage*, as introduced in [18]. We call these variable usage patterns *variable roles*.

**Fig. 2:**
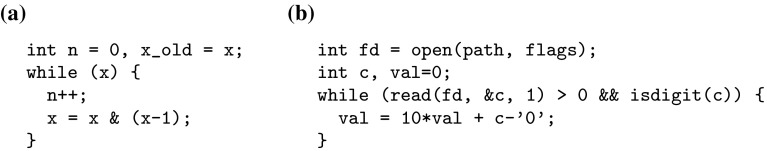
Different usage patterns of integer variables. **a** Bitvector, counter, linear. **b** Character, file descriptor

#### Example 1

Consider the C program in Fig. 2a, which computes the number of non-zero bits of variable x. In every loop iteration, a non-zero bit of x is set to zero and counter n is incremented. For a human reading the program, the statements n=0 and n++ in the loop body signal that n is a *counter*, and statement x = x & (x-1) indicates that x is a *bit vector*.

#### Example 2

Consider the program in Fig. 2b, which reads a decimal number from a text file and stores its numeric representation in variable val. Statement fd=open(path, flags) indicates that variable fd stores a *file descriptor* and statement isdigit(c) indicates that c is a *character*, because function isdigit() checks whether its parameter is a decimal digit character.

*Criteria for choosing roles* We define 27 variable roles and give their informal definition in Table 2. Our choice of roles is inspired by standard concepts used by programmers. In order to create the list of roles we inspected the source code of the cBench benchmark [11] and came up with a minimum set of roles such that every variable is assigned at least one role.

**Table 2:** List of variable roles with informal definitions

C type	Role name	Informal definition
int	ARRAY_INDEX	Occurs in an array subscript expression
	ALLOC_SIZE	Passed to a standard memory allocation function
	BITVECTOR	Used in a bitwise operation or assigned the result of a bitwise operation or a BITVECTOR variable
	BOOL	Assigned and compared only to 0, 1, the result of a bitwise operation, or a BOOL variable
	BRANCH_COND	Used in the condition of an if statement
	CHAR	Used in a library function which manipulates characters, or assigned a character literal
	CONST_ASSIGN	Assigned only literals or CONST_ASSIGN variables
	COUNTER	Changed only in increment/decrement statements
	FILE_DESCR	Passed to a library function which manipulates files
	INPUT	Assigned the result of an external function call or passed to it as a parameter by reference
	LINEAR	Assigned only linear combinations of LINEAR variables
	LOOP_BOUND	Used in a loop condition in a comparison operation, where it is compared to a LOOP_ITERATOR variable
	LOOP_ITERATOR	Occurs in loop condition, assigned in loop body
	MODE	Not used in comparison operations other than == and !=; assigned and compared to constant values only
	OFFSET	Added to or subtracted from a pointer
	SCALAR_INT	Scalar integer variable
	SYNT_CONST	Not assigned in the program (a global or an unused variable, or a formal parameter to a external function)
	THREAD_DESCR	Passed to a function of pthread library
	USED_IN_ARITHM	Used in addition/subtraction/multiplication/division
float	SCALAR_FLOAT	Scalar float variable
int*, float*	PTR_SCALAR	Pointer to a scalar value
struct_type*	PTR_STRUCT	Pointer to a structure
	PTR_STRUCT_PTR	Pointer to a structure which has a pointer field
	PTR_STRUCT_REC	Pointer to a recursively defined structure
	PTR_COMPL_STRUCT	Pointer to a recursively defined structure with more than one pointer, e.g. doubly linked lists
any_type*	HEAP_PTR	Assigned the result of a memory allocation function call
	PTR	Any pointer

Type $\textit{struct}\_\textit{type}$ stands for a C structure, $\textit{any}\_\textit{type}$ for an arbitrary C type

*Definition of roles* We define roles using data-flow analysis, an efficient fixed-point algorithm [1]. Our current definition of roles is control-flow insensitive, and the result of analysis is the set of variables ${ Res }^R$ which are assigned role *R*. For the exact definitions of variable roles, we refer the reader to [18].

#### Example 3

We describe the process of computing roles on the example of role LINEAR for the code in Fig. 2a. Initially, the algorithm assigns to ${ Res }^{\mathrm{LINEAR}}$ the set of all variables $\{\texttt {x},\texttt {x\_old},\texttt {n}\}$. Then it computes the greatest fixed point in three iterations. In iteration 1, variable x is removed, because it is assigned the non-linear expression x&(x-1), resulting in ${ Res }^{\mathrm{LINEAR}}=\{\texttt {x\_old},\texttt {n}\}$. In iteration 2, variable x_old is removed, because it is assigned variable x, resulting in ${ Res }^{\mathrm{LINEAR}}=\{\texttt {n}\}$. In iteration 3, ${ Res }^{\mathrm{LINEAR}}$ does not change, and the result of the analysis is ${ Res }^{\mathrm{LINEAR}}=\{\texttt {n}\}$.

#### Definition 1

(Variable role based metrics) For a given benchmark file *f*, we compute the mapping ${ Res }^R:{{ Roles }} \rightarrow 2^{{ Vars }}$ from variable roles to sets of program variables of *f*. We derive role metrics $m_R$ that represent the relative occurrence of each variable role $R \in { Roles }$:

1 $$\begin{aligned} m_R = \frac{|{ Res }^R|}{|{ Vars }|} \qquad R \in { Roles } \end{aligned}$$

### Loop pattern based metrics

The second set of program features we consider is a *classification of loops* in the program under verification, as introduced in [31]. Although undecidable in general, the ability to reason about bounds or termination of loops is highly useful for software verification: For example, it allows a tool to assert the (un)reachability of program locations after the loop, and to compute unrolling factors and soundness limits in the case of bounded model checking.

In [31] we present heuristics for loop termination. They are inspired by *definite iteration*, i.e. structured iteration over the elements of a finite set, such as an integer sequence or the elements of a data structure [37]. We first give a definition of definite iteration, which we call *FOR loops*, for the C programming language, as C does not have dedicated support for this concept. Then, we define *generalized FOR loops*, which capture some aspects of definite iteration and allow us to describe a majority of loops in our benchmarks. Table 3 gives an overview.

*FOR loops* We start by giving a loop pattern for a restricted set of bounded loops $\mathcal {L}^\mathrm {FOR}$, which is designed to capture definite iteration. We exploit that in many cases, local reasoning is powerful enough to decide termination of loops expressing definite iteration. This allows us to implement an efficient termination procedure using syntactic pattern matching and data-flow analysis.

**Fig. 3:**
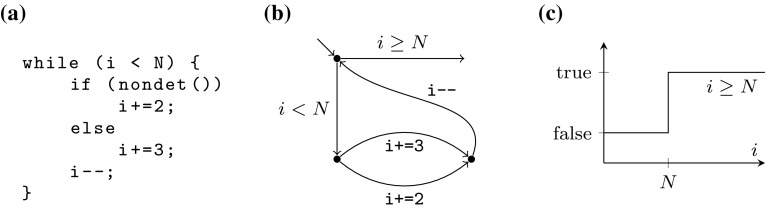
Example FOR loop $L \in \mathcal {L}^\mathrm {FOR}$. **a** Example loop source code. **b** The loop’s labeled transition system. **c** Representing function of the loop’s predicate

*Example* Consider the program shown in Fig. 3a. We show termination of the loop in a straight-forward manner: The value of i is changed by the loop, while the value of N is fixed. The loop’s condition induces a predicate $P(i): i \ge N$, guarding the edge leaving the loop (Fig. 3b). We show that during execution, *P*(*i*) eventually evaluates to true: The domain of *P* can be partitioned into two intervals $[-\infty , N)$ and $[N, \infty ]$, for which *P*(*i*) evaluates to false or true, respectively (Fig. 3c). As i is (in total) incremented during each iteration, we eventually have $i \in [N, \infty ]$, and thus *P*(*i*) holds and the loop terminates.

More formally, we find such a termination proof for a loop *L* in three steps:

1. For each variable *v* we establish the set of possible constant integral updates ${ Incs }(v)$ of *v* along all possible execution paths of a single iteration of *L*.
  In our example ${ Incs }(i) = \{1,2\}$.
2. We identify control flow edges *e* leaving the loop for which the corresponding $P_e(v)$ eventually evaluates to true under updates described by ${ Incs }(v)$.
  In our example there is a single such edge with predicate $P(i): i \ge N$. All values in ${ Incs }(i)$ are positive, thus *P*(*i*) eventually becomes true.
3. We impose a constraint to ensure $P_e(v)$ is evaluated in each iteration of *L*.
  In our example *P*(*i*) corresponds to the loop condition and this constraint is trivially satisfied.

**Table 3:** List of loop patterns with informal descriptions

Loop pattern	Empirical hardness	Informal definition
Syntactically bounded loops $\mathcal {L}^{\mathrm{bounded}}$	Easy	The number of executions of the loop body is bounded (considers outer control flow)
FOR loops $\mathcal {L}^{\mathrm{FOR}}$	Intermediate	The loop terminates whenever control flow enters it (disregards outer control flow)
Generalized FOR loops $\mathcal {L}^{\mathrm{FOR(*)}}$	Advanced	A heuristic derived from FOR loops by weakening the termination criteria. A good heuristic for termination
Hard loops $\mathcal {L}^{\mathrm{hard}}$	Hard	Any loop that is not classified as generalized FOR loop

We call a loop for which we obtain such a termination proof a *FOR loop* $L \in \mathcal {L}^{\mathrm{FOR}}$. In [31] we show how to efficiently implement these checks using syntactic pattern matching and data-flow analysis.

*Syntactically bounded loops* A stronger notion of termination considers a loop to be *bounded* if the number of executions of the loop body is bounded: A loop *L* is *syntactically bounded* $L \in \mathcal {L}^{\mathrm{bounded}}$ if and only if *L* itself and all its nesting (outer) loops are FOR loops: $L \in \mathcal {L}^{\mathrm{bounded}} \text { iff } \forall L_o \supseteq L \, . L_o \in \mathcal {L}^{\mathrm{FOR}}$.

*Generalized FOR loops* We impose strong constraints for classifying loops as $\mathcal {L}^{\mathrm{FOR}}$. In order to cover more loops, we systematically loosen these constraints and obtain a family of heuristics, which we call *generalized FOR loops* $\mathcal {L}^{\mathrm{FOR(*)}}$. We conjecture that this class still retains many features of FOR loops. We describe details of the constraint weakenings in [31]. Of the family of generalized FOR loop classes presented there, we only consider $\mathcal {L}^\mathrm {(\text {W}_{1}\text {W}_{2}\text {W}_{3})}$ for constructing the portfolio.

*Hard loops* Any loop not covered by $\mathcal {L}^{\mathrm{bounded}} \subseteq \mathcal {L}^{\mathrm{FOR}} \subseteq \mathcal {L}^{\mathrm{FOR(*)}}$ is classified as hard: Let $\mathcal {L}^{\mathrm{any}}$ be the set of all loops. Then $\mathcal {L}^{\mathrm{hard}} = \mathcal {L}^{\mathrm{any}} \setminus \mathcal {L}^{\mathrm{FOR(*)}}$.

#### Definition 2

(*Loop pattern based metrics*) For a given benchmark file *f*, we compute $\mathcal {L}^\mathrm {bounded}$, $\mathcal {L}^\mathrm {FOR}$, $\mathcal {L}^\mathrm {FOR(*)}$, $\mathcal {L}^\mathrm {hard}$, and the set of all loops ${ Loops }$. We derive loop metrics $m_{C}$ that represent the relative occurrence of each loop pattern *C*:

2 $$\begin{aligned} m_{C} = \frac{|\mathcal {L}^{C}|}{|{ Loops }|} \qquad C \in \{ \mathrm{bounded}, \mathrm{FOR}, \mathrm{FOR(*)}, \mathrm{hard} \} \end{aligned}$$

### Control flow based metrics

Complex control flow poses another challenge for program analysis. To measure the complexity of control flow, we introduce five additional metrics:

- For *intraprocedural control flow*, we count (a) the number of basic blocks in the control flow graph (CFG) $m_{\mathrm{cfgblocks}}$, and (b) the maximum indegree of any basic block in the CFG $m_\mathrm{maxindeg}$.
- To represent *indirect function calls*, we measure (a) the ratio $m_\mathrm{fpcalls}$ of call expressions taking a function pointer as argument, and (b) the ratio $m_\mathrm{fpargs}$ of parameters to such call expressions that have a function pointer type.
- Finally, to describe the use of *recursion*, we measure the number of direct recursive function calls $m_\mathrm{reccalls}$.

### Usefulness of our features for selecting a verification tool

In Sect. 4, we demonstrate that a portfolio built on top of these metrics performs well as a tool selector. In this section, we already give two reasons why we believe these metrics have predictive power in the software verification domain in the first place.

*Tool developer reports* The developer reports in the competition report for SV-COMP’15 [2], as well as tool papers (e.g. [10, 19]), give evidence for the relevance of our features for selecting verification tools: They mention language constructs, which—depending on whether they are fully, partially, or not modeled by a tool—constitute its strengths or weaknesses. We give a short survey of such language constructs in Table 1 and relate them to our features. For example, Predator is specifically built to deal with dynamic data structures (variable role PTR_STRUCT_REC) and pointer offsets (OFFSET), and CPAchecker does not model multi-threading (THREAD_DESCR) or support recursion (control flow feature $m_\mathrm{reccalls}$). For CBMC, unbounded loops (various loop patterns $\mathcal {L}^\mathrm {C}$) are an obstacle.

*Preliminary experiments* In addition, in previous work we have successfully used variable roles and loop patterns to deduce properties of verification tasks:

- In [18], we use *variable roles* to predict—for a given verification task—its category in SV-COMP’13.
- In [31], we show that *loop patterns* are good heuristics for identifying bounded loops.

These give further evidence for our claim that the features described above are useful in predicting properties of verification tasks.

## A portfolio solver for software verification

### Preliminaries on machine learning

In this section we introduce standard terminology from the machine learning community (see for example [7]).

#### Supervised machine learning

In supervised machine learning problems, we learn a *model* $M:\mathbb {R}^n \rightarrow \mathbb {R}$. The $\mathbf {x}_i \in \mathbb {R}^n$ are called *feature vectors*, measuring some property of the object they describe. The $y_i \in \mathbb {R}$ are called *labels*.

We learn model *M* by considering a set of labeled examples $X||\mathbf {y} = \{(\mathbf {x}_i, y_i)\}_{i=1}^N$. *M* is then used to predict the label of previously unseen inputs $\mathbf {x'} \notin X$.

We distinguish two kinds of supervised machine learning problems:

- *Classification* considers labels from a finite set $y \in \{1, \dots , C\}$. For $C=2$, we call the problem *binary classification*, for $C>2$ we speak of *multi-class classification*.
- *Regression* considers labels from the real numbers $y \in \mathbb {R}$.

#### Support vector machines

A *support vector machine* (SVM) [8, 16] is a binary classification algorithm that finds a hyperplane $\mathbf {w}\cdot \mathbf {x} + b = 0$ separating data points with different labels. We first assume that such a hyperplane exists, i.e. that the data is *linearly separable*:

Also called a *maximum margin classifier*, SVM learns a hyperplane that maximizes the gap $||\mathbf {w}||^{-1}$ (*margin*) between the hyperplane and the nearest data points with different labels. Maximizing the margin is formulated as

3 $$\begin{aligned} \text {minimize } ||\mathbf {w}|| \text { subject to } y_i(\mathbf {w}\cdot \mathbf {x}_i + b) \ge 1 \quad \text { for } i = 1, \dots , N \end{aligned}$$

which is usually encoded as the following quadratic programming problem:

4 $$\begin{aligned} \text {maximize } \sum \limits _{i=1}^N \alpha _i - \frac{1}{2}\sum \limits _{i,j=1}^{N} \alpha _i \alpha _j y_i y_j \mathbf {x}_i \cdot \mathbf {x}_j \quad \text { subject to } \quad \alpha _i \ge 0 \quad \text { and } \quad \sum \limits _{i=1}^N \alpha _i y_i = 0 . \end{aligned}$$

After computing the separating hyperplane on a set of labeled examples, a previously unseen feature vector $\mathbf {x'}$ is classified using function

5 $$\begin{aligned} M(\mathbf {x'}) = {{\mathrm{sgn}}}\left( \mathbf {w}\cdot \mathbf {x'} + b\right) . \end{aligned}$$

Thus *M* predicts the class of $\mathbf {x'}$ by computing on which side of the hyperplane it falls.

If the data is not linearly separable, e.g. due to outliers or noisy measurements, there are two orthogonal approaches that we both make use of in our portfolio solver:

*Soft-margin SVM.* Soft-margin SVM allows some data points to be misclassified while learning the hyperplane. For this, we associate a slack variable $\xi _i \ge 0$ with each data point $\mathbf {x}_i$, where

$$\begin{aligned} \xi _i = {\left\{ \begin{array}{ll}\text {the distance from the hyperplane} &{} \text {if }\mathbf {x}_i\text { is misclassified}\\ 0 &{} \text {otherwise}\end{array}\right. } . \end{aligned}$$

We thus replace Eq. 3 with the following equation:

6 $$\begin{aligned} \text {minimize } ||\mathbf {w}|| + C \sum \limits _{i=1}^N \xi _i \quad \text { subject to } y_i(\mathbf {w}\cdot \mathbf {x}_i + b) \ge 1-\xi _i \quad \text { for } i = 1, \dots , N \end{aligned}$$

and substitute $0 \le \alpha _i \le C$ for the constraint $\alpha _i \ge 0$ in Eq. 4. Parameter $C>0$ controls the trade-off between allowing misclassification and maximizing the margin.

*Kernel transformations* Another, orthogonal approach to data that is not linearly separable *in the input space*, is to transform it to a higher-dimensional *feature space* $\mathbb {H}$ obtained by a transformation $\phi : \mathbb {R}^n \rightarrow \mathbb {H}$. For example, 2-class data not linearly separable in $\mathbb {R}^2$ can be linearly separated in $\mathbb {R}^3$ if $\phi$ pushes points of class 1 above, and points of class 2 below some plane.

The quadratic programming formulation of SVM allows for an efficient implementation of this transformation: We define a *kernel function* $K(\mathbf {x}_i,\mathbf {x}_j) = \phi (\mathbf {x}_i) \cdot \phi (\mathbf {x}_j)$ instead of explicitly giving $\phi$, and replace the dot product in Eq. 4 with $K(\mathbf {x}_i,\mathbf {x}_j)$. An example of a non-linear kernel function is the *radial basis function* (RBF): $K(\mathbf {x}_i, \mathbf {x}_j)=\exp (-\gamma || \mathbf {x}_i - \mathbf {x}_j ||^2), ~\gamma > 0$.

For classifying unseen feature vectors $\mathbf {x'}$, we replace Eq. 5 with

7 $$\begin{aligned} M(\mathbf {x'}) = {{\mathrm{sgn}}}\left( \mathbf {w}\cdot \phi (\mathbf {x'}) + b\right) \quad \text { where } \mathbf {w} = \sum _{i=1}^N \alpha _i y_i \phi (\mathbf {x}_i) . \end{aligned}$$

#### Probabilistic classification

Probabilistic classification is a generalization of the classification algorithm, which searches for a function $M: \mathbb {R}^n \rightarrow \Pr (\mathbf {y})$, where $\Pr (\mathbf {y})$ is the set of all probability distributions over $\mathbf {y}$. $M(\mathbf {x'})$ then gives the probability ${{\mathrm{p}}}(y_i \mid \mathbf {x'}, X||\mathbf {y})$, i.e. the probability that $\mathbf {x'}$ actually has label $y_i$ given the model trained on $X||\mathbf {y}$. There is a standard algorithm for estimating class probabilities for SVM [41].

#### Creating and evaluating a model

The labeled set $X||\mathbf {y}$ used for creating (training) model *M* is called *training set*, and the set $X'$ used for evaluating the model is called *test set*. To avoid overly optimistic evaluation of the model, it is common to require that the training and test sets are disjoint: $X \cap X' = \emptyset$. A model which produces accurate results with respect to $||\mathbf {w}||$ for the training set, but results in a high error for previously unseen feature vectors $\mathbf {x'} \notin X$, is said to *overfit*.

#### Data imbalances

The training set $X||\mathbf {y}$ is said to be *imbalanced* when it exhibits an unequal distribution between its classes: $\exists y_i, y_j \in \mathbf {y} \text { . } {{{{\mathrm{num}}}(y_i)}/{{{\mathrm{num}}}(y_j)}} \sim 100$, where ${{\mathrm{num}}}(y)=|\{\mathbf {x}_i \in X \mid y_i=y\}|$, i.e. imbalances of the order 100:1 and higher. Data imbalances significantly compromise the performance of most standard learning algorithms [23].

A common solution for the imbalanced data problem is to use a *weighting function* ${{\mathrm{Weight}}}: X \rightarrow \mathbb {R}$ [25]. *SVM with weights* is a generalization of SVM, where we

8 $$\begin{aligned} \text {minimize } ||\mathbf {w}|| + C \sum \limits _{i=1}^N {{\mathrm{Weight}}}(\mathbf {x}_i) \xi _i . \end{aligned}$$

${{\mathrm{Weight}}}$ is usually chosen empirically.

#### Multi-class classification

SVM is by nature a binary classification algorithm. To tackle multi-class problems, we reduce an *n*-class classification problem to *n* binary classification problems: *One-vs.-rest* classification creates one model $M_i$ per class *i*, with the labeling function

$$\begin{aligned} M_i(\mathbf {x}) = {\left\{ \begin{array}{ll}1 &{} \text {if } M(\mathbf {x})=i\\ -1&{}\text {otherwise} \end{array}\right. } \end{aligned}$$

and the predicted value is calculated as $M(\mathbf {x})= { choose }\,\{i \mid M_i(\mathbf {x}) = 1\}$, where a suitable operator ${ choose }$ is used to select a single class from multiple predicted classes.

### The competition on software verification SV-COMP

In this section we give an overview of the competition’s setup. Detailed information about the competition is available on its website [15].

SV-COMP maintains a repository of verification tasks, on which the competition’s participants are tested:

#### Definition 3

(*Verification task*) We denote the set of all considered verification tasks as ${ Tasks }$. A verification task $v \in { Tasks }$ is described by a triple $v = (f, p, { type })$ of a C source file *f*, verification property *p* and property type ${ type }$. For SV-COMP’14 and ’15, ${ type }$ is either a label reachability check or a memory safety check (comprising checks for freedom of unsafe deallocations, unsafe pointer dereferences, and memory leaks). SV-COMP’16 adds the property types overflow and termination.

For each verification task, its designers define the expected answer, i.e. if property *p* holds on *f*:

#### Definition 4

(*Expected answer*) Function ${{\mathrm{ExpAns}}}: { Tasks }\rightarrow \{\textsf {true}, \textsf {false}\}$ gives the *expected answer* for task *v*, i.e. ${{\mathrm{ExpAns}}}(v) = \textsf {true}$ if and only if property *p* holds on *f*.

Furthermore, SV-COMP partitions the verification tasks ${ Tasks }$ into *categories*, a manual grouping by characteristic features such as usage of bitvectors, concurrent programs, linux device drivers, etc.

#### Definition 5

(*Competition category*) Let ${ Categories }$ be the set of competition categories. Let ${{\mathrm{Cat}}}: { Tasks }\rightarrow { Categories }$ define a partitioning of ${ Tasks }$, i.e. ${{\mathrm{Cat}}}(v)$ denotes the category of verification task *v*.

Finally, SV-COMP assigns a *score* to each tool’s result and computes weighted *category scores*. For example, the *Overall* SV-COMP score considers a meta category of all verification tasks, with each constituent category score normalized by the number of tasks in it. We describe and compare the scoring policies of recent competitions in Sect. 4.1. In addition, medals are awarded to the three best tools in each category. In case multiple tools have equal scores, they are ranked by runtime for awarding medals.

#### Definition 6

(*Score, category score,* *Overall* *score*) Let ${ score }_{t,v}$ denote the score of tool $t \in { Tools }$ on verification task $v \in { Tasks }$ calculated according to the rules of the respective edition of SV-COMP. Let ${{\mathrm{cat\_score}}}(t,c)$ denote the score of tool *t* on the tasks in category $c \in { Categories }$ calculated according to the rules of the respective edition of SV-COMP.

### Tool selection as a machine learning problem

In this section, we describe the setup of our portfolio solver $\mathcal {TP}$. We give formal definitions for modeling SV-COMP, describe the learning task as multi-class classification problem, discuss options for breaking ties between multiple tools predicted correct, present our weighting function to deal with data imbalances, and finally discuss implementation specifics.

#### Definitions

##### Definition 7

(*Verification tool*) We model the constituent verification tools as set ${ Tools }= \{1, 2, \dots , |{ Tools }|\}$ and identify each verification tool by a unique natural number $t \in { Tools }$.

##### Definition 8

(*Tool run*) The result of a run of tool *t* on verification task $v = (f, p, { type })$ is a triple

$$\begin{aligned} \langle { ans }_{t,v},{ runtime }_{t,v},{ memory }_{t,v}\rangle \end{aligned}$$

where ${ ans }_{t,v}\in \{\textsf {true}, \textsf {false}, \textsf {unknown}\}$ is the tool’s answer whether property *p* holds on file *f*, i.e.and ${ runtime }_{t,v}\in \mathbb {R}$ (resp. ${ memory }_{t,v}\in \mathbb {R}$) is the runtime (resp. memory usage) of tool *t* on task *v* in seconds (resp. megabytes).

##### Definition 9

(*Virtual best solver*) The *virtual best solver* (VBS) is an oracle that selects for each verification task the tool which gives the correct answer in minimal time.

#### Machine learning data

We compute feature vectors from the metrics introduced in Sect. 2 and the results of SV-COMP as follows:

For verification task $v = (f, p, { type })$ we define feature vector

$$\begin{aligned} \mathbf {x}(v)= (&m_\mathrm{ARRAY\_INDEX}(v),\dots ,m_\mathrm{PTR}(v),\\&m_\mathrm{bounded}(v),\dots ,m_\mathrm{hard}(v),\\&m_\mathrm{cfgblocks}(v),\dots ,m_\mathrm{reccalls}(v),\\&{ type }) \end{aligned}$$

where the $m_i(v)$ are our metrics from Sect. 2 computed on *f* and ${ type }\in \{0,1,2,3\}$ encodes if the property is reachability, memory safety, overflow, or termination.

We associate each feature vector $\mathbf {x}(v)$, with a label $t \in { Tools }$, such that *t* is the tool chosen by the virtual best solver for task *v*. In the following, we reduce the corresponding classification problem to $|{ Tools }|$ independent classification problems.

#### Formulation of the machine learning problem

For each tool $t \in { Tools }$, $\mathcal {TP}$ learns a model to predict whether tool *t* gives gives a correct or incorrect answer, or responds with “unknown”. Since the answer of a tool does not depend on the answers of other tools, $|{ Tools }|$ independent models (i.e., one per tool) give more accurate results and prevent overfitting.

We define labeling function $L_t(v)$ for tool *t* and task *v* as follows:

$$\begin{aligned} L_t(v) = {\left\{ \begin{array}{ll}1 &{} \quad \text {if } { ans }_{t,v}={{\mathrm{ExpAns}}}(v)\\ 2 &{}\quad \text {if } { ans }_{t,v}= \textsf {unknown}\\ 3&{}\quad \text {otherwise}\end{array}\right. }. \end{aligned}$$

I.e., $L_t(v) = 1$ if tool *t* gives the correct answer on *v*, $L_t(v) = 2$ if *t* answers $\textsf {unknown}$, and $L_t(v) = 3$ if *t* gives an incorrect answer. A tool can opt-out from a category, which we treat as if the tool had answered $\textsf {unknown}$ for all of the category’s verification tasks. Thus, for each tool *t*, we obtain training data $\{(\mathbf {x}(v), L_t(v))\}_{v \in { Tasks }}$ from which we construct model $M_t$.

*Tool selection based on predicted answer correctness.* Let operator ${ choose }:2^{ Tools }\rightarrow { Tools }$ select one tool from a set of tools ${ TPredicted } \subseteq { Tools }$ (we give concrete definitions of ${ choose }$ below). Given $|{ Tools }|$ predictions of the models $M_t, t \in { Tools }$ for a task *v*, the portfolio algorithm selects a single tool ${t}^\mathrm{best}$ as follows:

$$\begin{aligned} {t}^\mathrm{best}={\left\{ \begin{array}{ll} { choose }({{\mathrm{TCorr}}}(v)) &{}\quad \text {if } {{\mathrm{TCorr}}}(v) \ne \emptyset \\ { choose }({{\mathrm{TUnk}}}(v)) &{}\quad \text {if } {{\mathrm{TCorr}}}(v)=\emptyset \wedge {{\mathrm{TUnk}}}(v) \ne \emptyset \\ t^\mathrm{winner} &{}\quad \text {if } {{\mathrm{TCorr}}}(v)=\emptyset \wedge {{\mathrm{TUnk}}}(v) = \emptyset \end{array}\right. } \end{aligned}$$

where ${{\mathrm{TCorr}}}(v)$ and ${{\mathrm{TUnk}}}(v)$ are the sets of tools predicted to give the correct answer and respond with “unknown” on *v*, respectively:

$$\begin{aligned} {{\mathrm{TCorr}}}(v)&= \{t \in { Tools }\mid M_t(v) = 1\} \\ {{\mathrm{TUnk}}}(v)&= \{t \in { Tools }\mid M_t(v) = 2\} \end{aligned}$$

and $t^\mathrm{winner}$ is the *Overall* winner of the competition, e.g. UltimateAutomizer in SV-COMP’16.

#### Choosing among tools predicted correct

We now describe three alternative ways of implementing the operator ${ choose }$:

1. **Time:** $\mathcal {TP}^\mathbf{time}$. We formulate $|{ Tools }|$ additional regression problems: For each tool *t*, we use training data $\{(\mathbf {x}(v), { runtime }_{t,v}^\mathrm{norm})\}_{v \in { Tasks }}$ to obtain a model $M_t^\mathrm{time}(v)$ predicting runtime, where
  $$\begin{aligned} { runtime }_{t,v}^\mathrm{norm}= {{\mathrm{norm}}}({ runtime }_{t,v}, \{{ runtime }_{t',v'}\}_{t' \in { Tools }, v' \in { Tasks }}) \end{aligned}$$
  and ${{\mathrm{norm}}}$ normalizes to the unit interval:
  $$\begin{aligned} {{\mathrm{norm}}}(x, X) = \frac{x - \min ({ X })}{\max ({ X }) - \min ({ X })} . \end{aligned}$$
  The predicted value $M_t^\mathrm{time}(v)$ is the predicted runtime of tool *t* on task *v*. We define
  $$\begin{aligned} { choose }({ TPredicted }) = \mathop {\hbox {arg min}}\limits _{t \in { TPredicted }} M_t^\mathrm{time}(v) . \end{aligned}$$
2. **Memory:** $\mathcal {TP}^\mathbf{mem}$. Similar to $\mathcal {TP}^\mathrm{time}$, we formulate $|{ Tools }|$ additional regression problems: For each tool *t*, we use training data $\{(\mathbf {x}(v), { memory }_{t,v}^\mathrm{norm})\}_{v \in { Tasks }}$ to obtain a model $M_t^\mathrm{mem}(v)$ predicting memory, where
  $$\begin{aligned} { memory }_{t,v}^\mathrm{norm}= {{\mathrm{norm}}}({ memory }_{t,v}, \{{ memory }_{t',v'}\}_{t' \in { Tools }, v' \in { Tasks }}) . \end{aligned}$$
  We define
  $$\begin{aligned} { choose }({ TPredicted }) = \mathop {\hbox {arg min}}\limits _{t \in { TPredicted }} M_t^\mathrm{mem}(v) . \end{aligned}$$
3. **Class probabilities:** $\mathcal {TP}^\mathbf{prob}$. We define the operator
  $$\begin{aligned} { choose }({ TPredicted })=\mathop {\hbox {arg max}}\limits _{t \in { TPredicted }} P_{t,v} \end{aligned}$$
  where $P_{t,v}$ is the class probability estimate for $M_t(v)=1$, i.e. the probability that tool *t* gives the expected answer on *v*.

In Table 4 we present preliminary experiments comparing the ${ choose }$ operators for category *Overall* in the setup of SV-COMP’14. We consider the following criteria: the percentage of correctly and incorrectly answered tasks, SV-COMP score, runtime, memory usage, and the place in the competition^1^.

*Discussion.* $\mathcal {TP}^\mathrm{mem}$ and $\mathcal {TP}^\mathrm{time}$ clearly optimize the overall memory usage and runtime, respectively. At the same time, they fall behind $\mathcal {TP}^\mathrm{prob}$ with respect to the ratio of correct answers and SV-COMP score. Our focus here is on building a portfolio for SV-COMP, where tools are ranked by score. In the following we thus focus on the implementation of ${ choose }$ from $\mathcal {TP}^\mathrm{prob}$ and refer to it as $\mathcal {TP}$.

**Table 4:** Comparison of formulations of $\mathcal {TP}$, using different implementations of operator ${ choose }$

Setting	Correct/incorrect/unknown answers (%)	Score	Runtime (min)	Memory (GiB)	Place
$\mathcal {TP}^\mathrm{mem}$	88/2/10	1047	2819	390.2	3
$\mathcal {TP}^\mathrm{time}$	92/2/6	1244	920	508.4	1
$\mathcal {TP}^\mathrm{prob}$	94/1/5	1443	2866	618.1	1

Runtime shown here is de-normalized from the predicted (normalized) value defined above

#### Dealing with data imbalances

An analysis of the SV-COMP data shows that the labels $L_t(v)$ are highly imbalanced: For example, in SV-COMP’14 the label which corresponds to incorrect answers, $L_t(v) = 3$, occurs in less than 4% for every tool. The situation is similar for SV-COMP’15 and ’16. We therefore use SVM with weights, in accordance with standard practice in machine learning.

Given a task *v* and tool *t*, we calculate the weighting function ${{\mathrm{Weight}}}$ as follows:

$$\begin{aligned} {{\mathrm{Weight}}}(v,t) =&{{\mathrm{Potential}}}(v) \times {{\mathrm{Criticality}}}(v) \times \\&{{\mathrm{Performance}}}(t,{{\mathrm{Cat}}}(v)) \times {{\mathrm{Speed}}}(t,{{\mathrm{Cat}}}(v)) . \end{aligned}$$

We briefly give informal descriptions of functions ${{\mathrm{Potential}}}$, ${{\mathrm{Criticality}}}$, ${{\mathrm{Performance}}}$, ${{\mathrm{Speed}}}$ before defining them formally:

- $\mathbf {Potential}(v)$ describes how important predicting a correct tool for task *v* is, based on its score potential. E.g., unsafe tasks (${{\mathrm{ExpAns}}}= \textsf {false}$) have more points deducted for incorrect answers than safe (${{\mathrm{ExpAns}}}= \textsf {true}$) tasks, thus their score potential is higher.
- $\mathbf {Criticality}(v)$ captures how important predicting a correct tool is, based on how many tools give a correct answer. Intuitively, this captures how important an informed decision about task *v*, as opposed to a purely random guess, is.
- $\mathbf {Performance}(t,c)$ describes how well tool *t* does on category *c* compared to the category winner.
- $\mathbf {Speed}(t,c)$ describes how fast tool *t* solves tasks in category *c* compared to the fastest tool in the category.

More formally,

$$\begin{aligned} {{\mathrm{Potential}}}(v)= {{\mathrm{score}}}_\mathrm{max}(v)-{{\mathrm{score}}}_\mathrm{min}(v) \end{aligned}$$

where ${{\mathrm{score}}}_\mathrm{max}(v)$ and ${{\mathrm{score}}}_\mathrm{min}(v)$ are the maximal and minimal possible scores for task *v*, respectively. For example, in the setup of SV-COMP’14, if *v* is safe, then ${{\mathrm{score}}}_\mathrm{max}(v)=2$ and ${{\mathrm{score}}}_\mathrm{min}(v)=-8$.

$$\begin{aligned} {{\mathrm{Criticality}}}(v)=|\{t \in { Tools }\mid { ans }_{t,v}= {{\mathrm{ExpAns}}}(v)\}|^{-1} \end{aligned}$$

is inversely proportional (subject to a constant factor) to the probability of randomly choosing a tool which gives the correct answer.^2^

$$\begin{aligned} {{\mathrm{Performance}}}(t,c)=\frac{{{\mathrm{cat\_score}}}(t,c) - {{\mathrm{cat\_score}}}_\mathrm{min}(c)}{{{\mathrm{cat\_score}}}(t^{ cbest },c) - {{\mathrm{cat\_score}}}_\mathrm{min}(c)} \end{aligned}$$

is the ratio of SV-COMP scores of tool *t* and the category winner $t^{ cbest }$ on tasks from category *c*, where

$$\begin{aligned} t^{ cbest }&= \mathop {\hbox {arg max}}\limits _{t_i \in { Tools }} {{\mathrm{cat\_score}}}(t_i,c) \\ {{\mathrm{cat\_score}}}(t,c)&= \sum \limits _{\{v \in { Tasks }\mid {{\mathrm{Cat}}}(v)=c\}}score_{t,v} \\ {{\mathrm{cat\_score}}}_\mathrm{min}(c)&= \sum \limits _{\{v \in { Tasks }\mid {{\mathrm{Cat}}}(v)=c\}} {{\mathrm{score}}}_\mathrm{min}(v) \end{aligned}$$

and $score_{t,v}$ is the SV-COMP score of tool *t* on task *v*.

$$\begin{aligned} {{\mathrm{Speed}}}(t,c)=\frac{\ln \,{{\mathrm{rel\_time}}}(t,c)}{\ln \,{{\mathrm{rel\_time}}}(t^{ cfst },c)} \end{aligned}$$

is the ratio of orders of magnitude of normalized total runtime of tool *t* and of the fastest tool $t^{ cfst }$ in category *c*, where

$$\begin{aligned} {{\mathrm{rel\_time}}}(t,c)&=\frac{{{\mathrm{cat\_time}}}(t,c)}{\sum _{t_i \in { Tools }}{{\mathrm{cat\_time}}}(t_i,c)}\\ t^{ cfst }&=\mathop {\hbox {arg min}}\limits _{t_i \in { Tools }}{{{\mathrm{cat\_time}}}(t_i,c)}\\ {{\mathrm{cat\_time}}}(t,c)&= \sum \limits _{\{v \in { Tasks }\mid {{\mathrm{Cat}}}(v)=c\}}{ runtime }_{t,v}. \end{aligned}$$

**Fig. 4:**
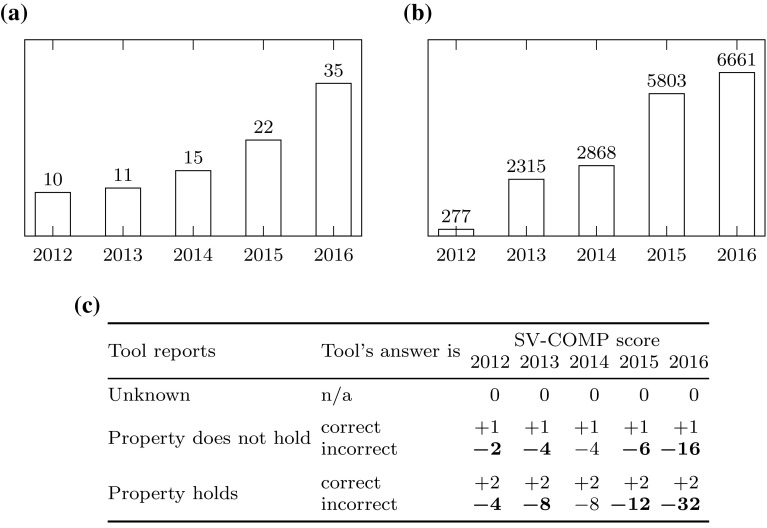
SV-COMP over the years: number of participants, number of verification tasks, scoring policy. **a** Number of participants in SV-COMP over the years. **b** Number of verification tasks in SV-COMP over the years. **c** Scoring policies of SV-COMP 2014, 2015, and 2016. Changing scores are shown in bold

#### Implementation of $\mathcal {TP}$

Finally, we discuss details of the implementation of $\mathcal {TP}$. We use the SVM machine learning algorithm with the RBF kernel and weights as implemented in the LIBSVM library [9]. To find optimal parameters *C* for soft-margin SVM and $\gamma$ for the RBF kernel, we do exhaustive search on the grid, as described in [24].

## Experimental results

### SV-COMP 2014 versus 2015 versus 2016

*Candidate tools and verification tasks.* Considering the number of participating tools, SV-COMP is a success story: Figure 4a shows the increase of participants over the years. Especially the steady increase in the last 2 years is a challenge for our portfolio, as the number of machine learning problems (cf. Sect. 3.3) increases. As Fig. 4b shows, also the number of verification tasks used in the competition has increased steadily.

*Scoring.* As described in Sect. 3.2, SV-COMP provides two metrics for comparing tools: score and medal counts. As Table 4c shows, the scoring policy has constantly changed (the penalties for incorrect answers were increased). At least for 2015, this was decided by a close jury vote [38]. We are interested how stable the competition ranks are under different scoring policies. Table 5 gives the three top-scoring tools in *Overall* and their scores in SV-COMP, as well as the top-scorers of each year if the scoring policy of other years had been applied:

Clearly, the scoring policy has a major impact on the competition results: In the latest example of SV-COMP’16, UltimateAutomizer wins SV-COMP’16 with the original scoring policy applied, but is not even among the three top-scorers if the policies of 2015 or 2014 are applied.

Given that SV-COMP score and thus also medal counts are rather volatile, we introduce *decisiveness-reliability plots* (DR-plots) in the next section to complement our interpretation of the competition results.

**Table 5:** *Overall* competition ranks for SV-COMP’14–’16 under the scoring policies of SV-COMP’14–’16

Year competition scoring		$1{\mathrm{st}}$ place (score)	$2{\mathrm{nd}}$ place (score)	$3{\mathrm{rd}}$ place (score)
2014	2014	CBMC (3501)	CPAchecker (2987)	LLBMC (1843)
	2015	CBMC (3052)	CPAchecker (2961)	LLBMC (1788)
	2016	CPAchecker (2828)	LLBMC (1514)	UFO (1249)
2015	2014	CPAchecker (5038)	SMACK (3487)	CBMC (3473)
	2015	CPAchecker (4889)	SMACK (3168)	UAutomizer (2301)
	2016	CPAchecker (4146)	SMACK (1573)	PredatorHQ (1169)
2016	2014	CBMC (6669)	CPA-Seq (5357)	ESBMC (5129)
	2015	CBMC (6122)	CPA-Seq (5263)	ESBMC (4965)
	2016	UAutomizer (4843)	CPA-Seq (4794)	SMACK (4223)

**Fig. 5:**
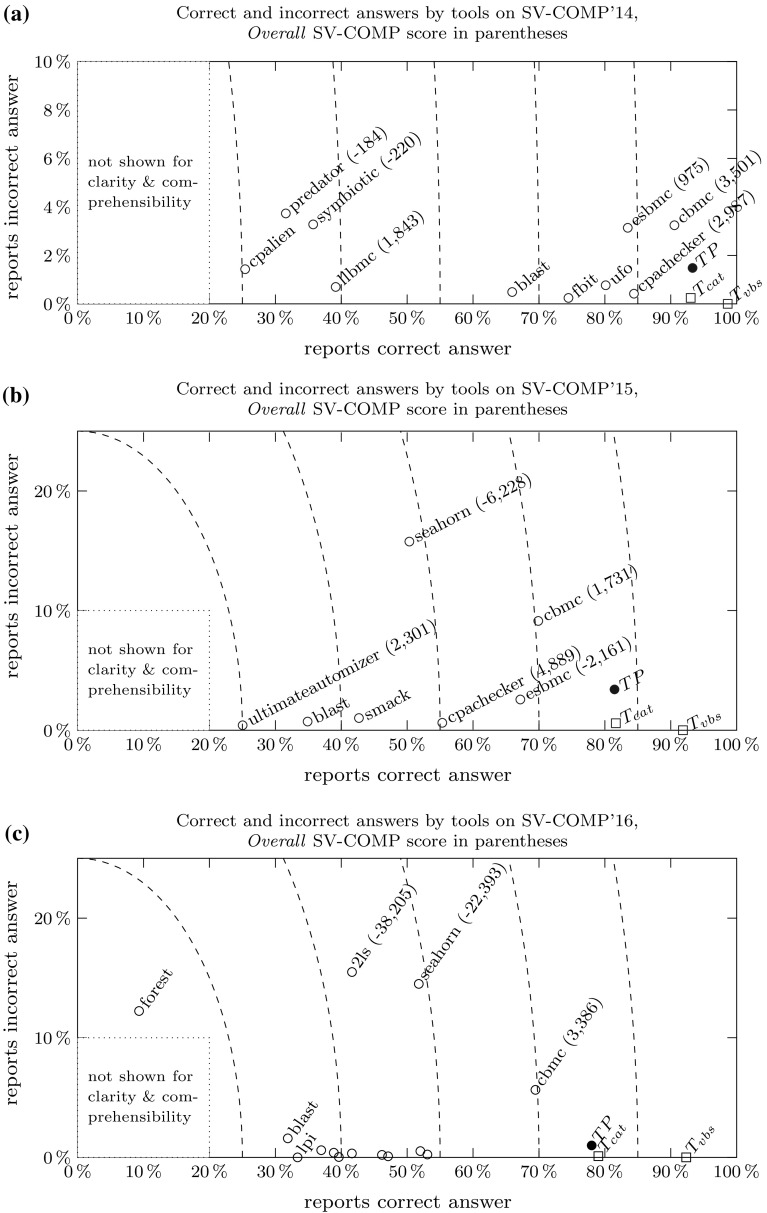
Decisiveness-reliability plots for SV-COMP’14–’16. The *horizontal axis* gives the percentage of correct answers *c*, the *vertical axis* the number of incorrect answers *i*. *Dashed lines* connect points of equal decisiveness $c+i$. The *Overall* SV-COMP score is given (if available) in parentheses. **a** Decisiveness-reliability plot for SV-COMP’14. **b** Decisiveness-reliability plot for SV-COMP’15. **c** Decisiveness-reliability plot for SV-COMP’16

### Decisiveness-reliability plots

To better understand the competition results, we create scatter plots where each data point $\mathbf {v} = (c,i)$ represents a tool that gives $c\%$ correct answers and $i\%$ incorrect answers. Figure 5 shows such plots based on the verification tasks in SV-COMP’14, ’15, and ’16. Each data point marked by an unfilled circle $\circ$ represents one competing tool. The rectilinear distance $c+i$ from the origin gives a tool’s *decisiveness*, i.e. the farther from the origin, the fewer times a tool reports “unknown”. The angle enclosed by the horizontal axis and $\mathbf {v}$ gives a tool’s *(un)reliability*, i.e. the wider the angle, the more often the tool gives incorrect answers. Thus, we call such plots *decisiveness-reliability plots* (DR-plots).

*Discussion.* Figure 5 shows DR-plots for the verification tasks in SV-COMP’14–’16:

- *For 2014* (Fig. 5a), all the tools are performing quite well on soundness: none of them gives more than 4% of incorrect answers. CPAchecker, ESBMC and CBMC are highly decisive tools, with more than 83% correct answers.
- *For 2015* (Fig. 5b), the number of verification tasks more than doubled, and there is more variety in the results: We see that very reliable tools (BLAST, SMACK, and CPAchecker) are limited in decisiveness—they report “unknown” in more than 40% of cases. The bounded model checkers CBMC and ESBMC are more decisive at the cost of giving up to 10% incorrect answers.
- *For 2016* (Fig. 5c), there is again a close field of very reliable tools (CPAchecker, SMACK, and UltimateAutomizer) that give around 50% of correct answers and almost no incorrect answers. Bounded model checker CBMC is still highly decisive, but gives 6% of incorrect answers.

We also give *Overall* SV-COMP scores (where applicable) in parentheses. Clearly, tools close together in the DR-plot not necessarily have similar scores because of the different score weights prescribed by the SV-COMP scoring policy.

Referring back to Fig. 5a–c, we also show the theoretic strategies $T_\mathrm{cat}$ and $T_\mathrm{vbs}$ marked by a square $\square$: Given a verification task *v*, $T_\mathrm{cat}$ selects the tool winning the corresponding competition category ${{\mathrm{Cat}}}(v)$. $T_\mathrm{vbs}$ is the *virtual best solver* (VBS) and selects for each verification task the tool which gives the correct answer in minimal time. Neither $T_\mathrm{cat}$ nor $T_\mathrm{vbs}$ can be built in practice: For $T_\mathrm{cat}$, we would need to know competition category ${{\mathrm{Cat}}}(v)$ of verification task *v*, which is withheld from the competition participants. For $T_\mathrm{vbs}$, we would need an oracle telling us the tool giving the correct answer in minimal time. Thus any practical approach must be a heuristic such as the portfolio described in this work.

However, both strategies illustrate that combining tools can yield an almost perfect solver, with $\ge 90\%$ correct and 0% incorrect answers. (Note that these figures may give an overly optimistic picture—after all the benchmarks are supplied by the competition participants.) The results for $T_\mathrm{vbs}$ compared to $T_\mathrm{cat}$ indicate that leveraging not just the category winner, but making a per-task decision provides an advantage both in reliability and decisiveness. A useful portfolio would thus lie somewhere between CPAchecker, CBMC, $T_\mathrm{cat}$, and $T_\mathrm{vbs}$, i.e. improve upon the decisiveness of constituent tools while minimizing the number of incorrect answers.

### Evaluation of our portfolio solver

We originally implemented the machine learning-based portfolio $\mathcal {TP}$ for SV-COMP’14 in our tool *Verifolio* [40]. When competition results for SV-COMP’15 became available, we successfully evaluated the existing techniques on the new data, and described our results in [17]. For SV-COMP’16, we reused the portfolio construction published there to compute the additional results in this paper. We present these both in terms of the traditional metrics used by the competition (SV-COMP score and medals) and $\mathcal {TP}$’s placement in DR-plots:

*Setup* For our experiments we did not rebuild the infrastructure of SV-COMP, but use numeric results from held competitions to compare our portfolio approach against other tools. Following a standard practice in machine learning [7], we randomly split the verification tasks of SV-COMP’${ year }$ into a training set ${ train }_{ year }$ and a test set ${ test }_{ year }$ with a ratio of 60:40. We train $\mathcal {TP}$ on ${ train }_{ year }$ and evaluate it on ${ test }_{ year }$ by comparing it against other tools’ results on ${ test }_{ year }$. As the partitioning into training and test sets is randomized, we conduct the experiment 10 times and report the arithmetic mean of all figures. Tables 6a–c show the *Overall* SV-COMP scores, runtimes and medal counts. The DR-plots in Fig.  5a–c show the portfolio marked by a filled circle $\bullet$.

**Fig. 6:**
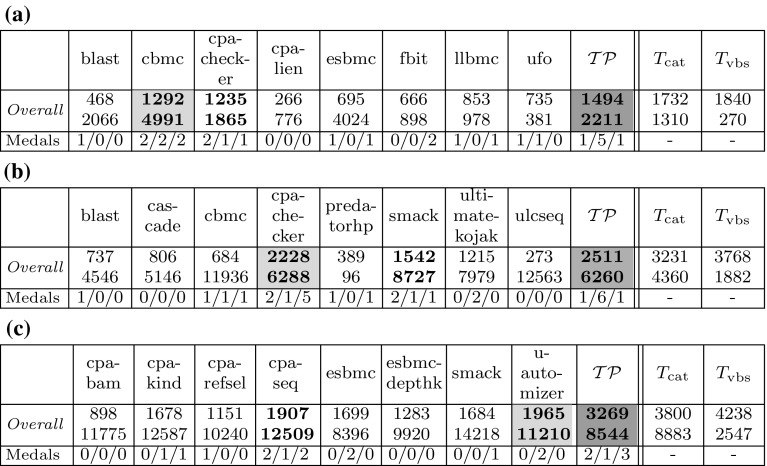
Experimental results for the eight best competition participants in *Overall* (for comprehensive result tables, cf. Tables 6–8), plus our portfolio $\mathcal {TP}$ on *random subsets* of SV-COMP, given as arithmetic mean of 10 experiments on the resp. test sets ${ test }_{ year }$. The two last columns show the idealized strategies $T_\mathrm{cat}$, $T_\mathrm{vbs}$ (not competing, for comparison only). The *first row* shows the *Overall* SV-COMP score and beneath it the runtime in minutes. We highlight the gold, silver, and bronze medal in *dark gray*, *light gray* and *white+bold font*, respectively. The *second row* shows the number of *gold/silver/bronze* medals won in individual categories. **a** *Overall* SV-COMP score, runtime and medal counts for SV-COMP’14. **b** *Overall* SV-COMP score, runtime and medal counts for SV-COMP’15. **c** *Overall* SV-COMP score, runtime and medal counts for SV-COMP’16

**Table 6:** Experimental results for the competition participants, plus our portfolio $\mathcal {TP}$ on *random subsets* of SV-COMP’14, given as arithmetic mean of 10 experiments on the resp. test sets ${ test }_{ year }$

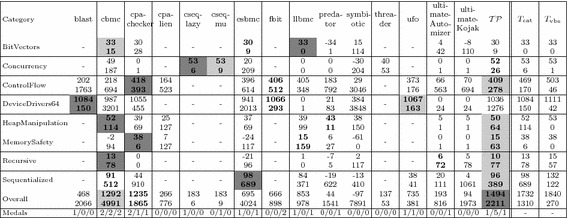	

The two last columns show the idealized strategies $T_\mathrm{cat}$, $T_\mathrm{vbs}$ (not competing, for comparison only). The first rows show the resp. SV-COMP score and beneath it the runtime in minutes. We highlight the gold, silver, and bronze medal in dark gray, light gray and white $+$ bold font, respectively. The last row shows the number of gold/silver/bronze medals won in individual categories

*Discussion* First, we discuss our results in terms of *Overall* SV-COMP score and medals:

- For SV-COMP’14 (Figure 6a), our portfolio $\mathcal {TP}$ overtakes the original *Overall* winner CBMC with 16% more points. It wins a total of seven medals (1/5/1 gold/silver/bronze) compared to CBMC’s six medals (2/2/2).
- For SV-COMP’15 (Figure 6b), $\mathcal {TP}$ is again the strongest tool, collecting 13% more points than the original *Overall* winner CPAchecker. Both CPAchecker and $\mathcal {TP}$ collect 8 medals, with CPAchecker’s 2/1/5 against $\mathcal {TP}$’s 1/6/1.
- For SV-COMP’16 (Figure 6c), $\mathcal {TP}$ beats the original *Overall* winner UltimateAutomizer, collecting 66% more points. $\mathcal {TP}$ collects 6 medals, compared to the original winner UltimateAutomizer with 2 medals (0/2/0) and the original runner-up CPA-Seq with 5 medals (2/1/2).

Second, we discuss the DR-plots in Figure 5a–c. Our portfolio $\mathcal {TP}$ positions itself between CBMC, CPAchecker and the theoretic strategies $T_\mathrm{cat}$ and $T_\mathrm{vbs}$. Furthermore, $\mathcal {TP}$ falls halfway between the concrete tools and idealized strategies. We think this is a promising result, but there is still room for future work. Here we invite the community to contribute further feature definitions, learning techniques, portfolio setups, etc. to enhance this approach.

In the following we discuss three aspects of $\mathcal {TP}$’s behavior in greater detail: The runtime overhead of feature extraction, diversity in the tools chosen by $\mathcal {TP}$, and cases in which $\mathcal {TP}$ selects a tool that gives the wrong answer.

**Fig. 7:**
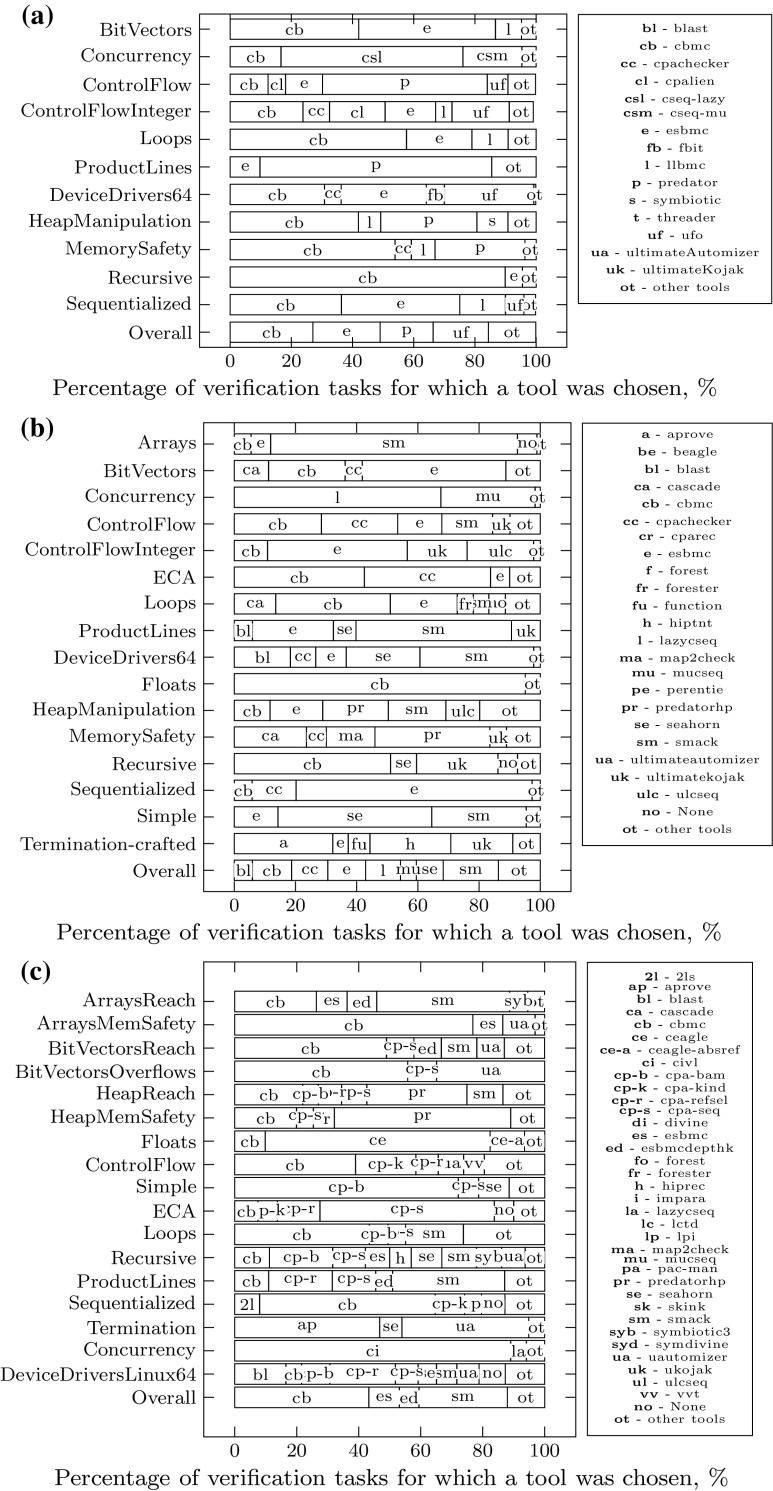
Compositionality of the portfolio $\mathcal {TP}$: Constituent tools selected per competition category. Tools selected in less than 5% of cases are summarized under label “other tools”. **a** Tools selected by $\mathcal {TP}$ for SV-COMP’14. **b** Tools selected by $\mathcal {TP}$ for SV-COMP’15. **c** Tools selected by $\mathcal {TP}$ for SV-COMP’16

#### Constituent verifiers employed by our portfolio

Our results could suggest that $\mathcal {TP}$ implements a trade-off between CPAchecker’s conservative-and-sound and CBMC’s decisive-but-sometimes-unsound approach. Contrarily, our experiments show that significantly more tools get selected by our portfolio solver (cf. Fig. 7a–c). Additionally, we find that our approach is able to select domain-specific solvers: For example, in the Concurrency category, $\mathcal {TP}$ almost exclusively selects variants of CSeq (and for 2016 also CIVL), which are specifically aimed at concurrent problems.

#### Wrong predictions

We manually investigated cases of wrong predictions made by the portfolio solver. We identify i. imperfect tools and ii. data imbalances as the two main reasons for bad predictions. In the following, we discuss them in more detail:

*Imperfect tools.* In SV-COMP, many unsafe (${{\mathrm{ExpAns}}}(v) = \textsf {false}$) benchmarks are manually derived from their safe (${{\mathrm{ExpAns}}}(v') = \textsf {true}$) counterparts with minor changes (e.g. flipping a comparison operator). Two such files have similar or even the same metrics ($\mathbf {x}(v) \approx \mathbf {x}(v')$), but imperfect tools don’t solve or fail to solve both of them ($L_t(v) \ne L_t(v')$). In particular, tools in SV-COMP are

- **unsound:** for example, in SV-COMP’16 the benchmarks 
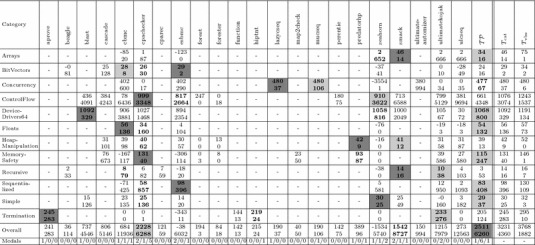
 differ in a single comparison operator, namely equality is changed to inequality. Tool BLAST solves the unsafe task correctly, and the safe one incorrectly (i.e. gives the same answer for both).
- **buggy:** similarly to above, in SV-COMP’16 benchmarks 
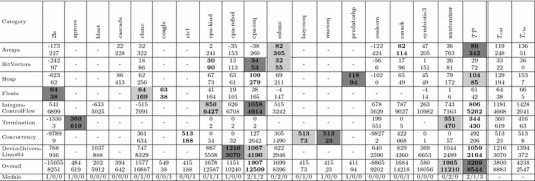
 differ in a single comparison operator. The tool Forest solves the safe task correctly, and crashes on the unsafe one.
- **incomplete:** the benchmarks 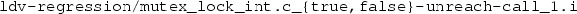, also taken from SV-COMP’16, differ in a single function call, namely mutex_unlock() is changed to mutex_lock(). The tool CASCADE correctly solves the safe benchmark, and answers unknown for the unsafe one.

This is unfortunate, as machine learning builds on the following assumption: Given two feature vectors $\mathbf {x}$ and $\mathbf {x'}$ with actual labels *y* and $y'$, if $\mathbf {x} \approx \mathbf {x'}$ (where approximate equality $\approx$ is defined by the machine learning procedure), then $y = y'$. This assumption is violated in the cases illustrated above.

*Counter-measures:* In all cases, our metrics do not distinguish the given benchmark pairs. To mitigate these results, the obvious solution is to improve the participating tools. To solve the issue on the side of our portfolio, we believe more expensive analyses would have to be implemented for feature extraction. However, these analyses would i. be equivalent to correctly solving the verification problem directly and ii. increase the overhead spent on feature extraction. A practical portfolio is thus limited by the inconsistencies exhibited by its individual tools.

*Data imbalances* In our training data we can find feature vectors on which, for a given tool *t*, e.g. the number of correct answers noticeably outweighs the number of incorrect answers. This corresponds to the problem of data imbalances (cf. Sect. 3.1.5), which leads to the following bias in machine learning: For a verification tool that is correct most of the time, machine learning prefers the error of predicting that the tool is correct (when in fact incorrect) over the error that a tool is incorrect (when in fact correct). In other words, “good” tools are predicted to be even “better”.

*Counter-measures:* As described in Sect. 3.1.5, the standard technique to overcome data imbalances are weighting functions. Discovering data imbalances and countering multiple of them in a single weighting function is a hard problem. Our weighting function (cf. Sect. 3.3.5) mitigates this issue by compensating several imbalances that we identified in our training data, and was empirically tuned to improve results while staying general.

#### Overhead of feature extraction

By construction, our portfolio incurs an overhead for feature extraction and prediction before actually executing the selected tool. In our experiments, we measured this overhead to take a median time of $\tilde{x}_\mathrm{features} = 0.5$ s for feature extraction and $\tilde{x}_\mathrm{prediction} = 0.5$ s for prediction. We find this overhead to be negligible, when compared to verification time. For example, the *Overall* winner of SV-COMP’16, UltimateAutomizer, exhibits a median verification time of $\tilde{x}^\mathrm{ua}_\mathrm{verif} = 24.9$ s computed over all tasks in SV-COMP’16.

Note that these numbers are not directly comparable, as $\tilde{x}^\mathrm{ua}_\mathrm{verif}$ stems from the SV-COMP results on the SV-COMP cluster, whereas $\tilde{x}_t$ for $t \in \{\text {features}, \text {prediction}\}$ was measured during our own experiments on a different system.

**Table 7:** Experimental results for the competition participants, plus our portfolio $\mathcal {TP}$ on *random subsets* of SV-COMP’15, given as arithmetic mean of 10 experiments on the resp. test sets ${ test }_{ year }$

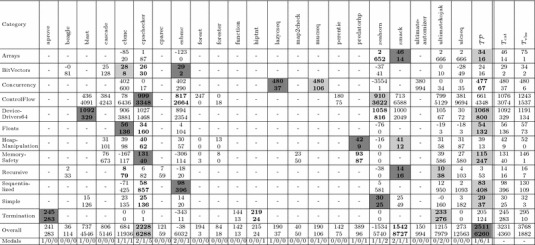	

The two last columns show the idealized strategies $T_\mathrm{cat}$, $T_\mathrm{vbs}$ (not competing, for comparison only). The first rows show the resp. SV-COMP score and beneath it the runtime in minutes. We highlight the gold, silver, and bronze medal in dark gray, light gray and white $+$ bold font, respectively. The last row shows the number of gold/silver/bronze medals won in individual categories

**Table 8:** Experimental results for the competition participants, plus our portfolio $\mathcal {TP}$ on *random subsets* of SV-COMP’16, given as arithmetic mean of 10 experiments on the resp. test sets ${ test }_{ year }$

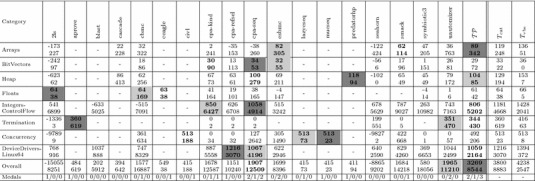	

The two last columns show the idealized strategies $T_\mathrm{cat}$, $T_\mathrm{vbs}$ (not competing, for comparison only). The first rows show the resp. SV-COMP score and beneath it the runtime in minutes. We highlight the gold, silver, and bronze medal in dark gray, light gray and white $+$ bold font, respectively. The last row shows the number of gold/silver/bronze medals won in individual categories.

For readability, we omit tools that did not win any medals in the original competition

## Related work

Portfolio solvers have been successful in combinatorially cleaner domains such as SAT solving [27, 35, 42], quantified boolean satisfiability (QSAT) [32, 33, 36], answer set programming (ASP) [20, 29], and various constraint satisfaction problems (CSP) [21, 28, 30]. In contrast to software verification, in these areas constituent tools are usually assumed to be correct.

A machine-learning based method for selecting model checkers was previously introduced in [39]. Similar to our work, the authors use SVM classification with weights (cf. Sect. 3.1). Our approach is novel in the following ways:

1. The results in [39] are not reproducible because i. the benchmark is not publicly available, ii. the verification properties are not described, and iii. the weighting function—in our experience crucial for good predictions—is not documented.
2. We demonstrate the continued viability of our approach by applying it to new results of recent SV-COMP editions.
3. We use a larger set of verification tools (35 tools vs. 3). Our benchmark is not restricted to device drivers and is >10 times larger (56 MLOC vs. 4 MLOC in [39]).
4. In contrast to structural metrics of [39] our metrics are computed using data-flow analysis. Based on tool designer reports (Table 1) we believe that they have superior predictive power. Precise comparison is difficult due to non-reproducibility of [39].

## Conclusion

In this paper we demonstrate the importance of software metrics to predict and explain the performance of verification tools. As software verification is a highly multidisciplinary effort and tools have highly diverse strengths and weaknesses, we believe that portfolio solving is a relevant research direction, well worthy of a competition track in its own right. In such a competition, a part of the benchmarks could be hidden from participating tools to prevent overfitting.

In future work, we also envision the use of software metrics for self-evaluation, i.e. better and more systematic descriptions of the benchmarks that accompany research papers in verification.

## Footnotes

1 In our previous work [17] there was a discrepancy in the runtime of $\mathcal {TP}^\mathrm{time}$ due to a bug in the code of $\mathcal {TP}^\mathrm{time}$. Table 4 shows the corrected result. We thank the anonymous reviewer for pointing out this issue.

2 We slightly adapt the formula of ${{\mathrm{Performance}}}$ compared to [17], such that ${{\mathrm{Weight}}}$ is always positive.
